# Risk factors for fast-growing lung cancers detected on chest CT: a retrospective cohort study

**DOI:** 10.3389/fonc.2026.1789510

**Published:** 2026-03-20

**Authors:** Linxin Liu, Rui Zhang, Renjie Xu, Bojiang Chen, Yi Lei, Weimin Li

**Affiliations:** 1 Department of Pulmonary and Critical Care Medicine, West China Hospital, Sichuan University, Chengdu, Sichuan, China; 2 State Key Laboratory of Respiratory Health and Multimorbidity, West China Hospital, Sichuan University, Chengdu, Sichuan, China; 3 General Practice Medical Center, General Practice Research Institute, West China Hospital, Sichuan University, Chengdu, Sichuan, China; 4 Institute of Respiratory Health, Frontiers Science Center for Disease-related Molecular Network, West China Hospital, Sichuan University, Chengdu, Sichuan, China; 5 Precision Medicine Center, Precision Medicine Key Laboratory of Sichuan Province, West China Hospital, Sichuan University, Chengdu, Sichuan, China

**Keywords:** fast-growing, lung cancer, risk factor, solid density, TP53 mutation

## Abstract

**Background:**

Chest CT follow-up is frequently used before a lung cancer is diagnosed. The current study aims to explore the risk factors for the fast-growing lung cancers through a retrospective cohort study.

**Methods:**

This study selected eligible participants from a cohort of 39799 patients pathologically diagnosed with primary lung cancer at West China Hospital of Sichuan University from 2009 to 2020. Ultimately, 1693 patients were included, who were followed up with at least two chest CT images available before diagnosis. The volume/mass doubling time (VDT/MDT) of all lung cancers were calculated, and a fast-growing lung cancer was defined if the VDT/MDT was less than 400 days. Multivariate logistic regression analysis was used to explore risk factors associated with fast-growing lung cancers in the overall population, as well as in the solid and subsolid subgroups.

**Results:**

Among the 1693 patients (median age 56 years, 37% male, 21% ever smokers, 27% with solid density), 302 (18%) were classified as having fast-growing lung cancer. In the subgroup analysis of solid versus subsolid groups, fast-growing lung cancer accounted for 41% and 9.4%, respectively. In the overall population, risk factors independently associated with rapid growth included solid density, male sex, smoking history, personal and family history of malignancy. In the solid subgroup, the risk factors were male sex and smoking history, while in the subsolid subgroup, only smoking history was significant. Additionally, analysis of 128 patients with a 56-gene panel (18% with rapid growth) identified TP53 as an independent risk factor for fast growth.

**Conclusions:**

This study found risk factors associated with fast-growing lung cancers, helping to identify patients at high risk of disease progression.

## Introduction

According to GLOBOCAN data, in 2022, there were nearly 2.5 million new cases of lung cancer worldwide (accounting for 12.4% of global cancer cases), and approximately 1.8 million deaths (constituting 18.7% of global cancer mortality), making it the cancer with the highest incidence and mortality rates (1). In China, in 2022, there were approximately 1,060,600 newly diagnosed cases of lung cancer (accounting for 22.0% of all malignant tumors), with approximately 733,300 deaths (accounting for 28.5% of all cancer-related deaths). Lung cancer ranked first in both incidence and mortality rates among malignant tumors in both males and females in China (2). Current research indicates that traditional risk factors such as smoking (3), air pollution, occupational exposure to carcinogens (4), and genetic mutations play significant roles in the initiation of lung cancer. However, there is a lack of systematic studies on the risk factors associated with fast progression of lung cancer from initial lesions to invasive stages. In routine clinical practice, notably, some lung cancer patients experience rapid disease deterioration within months after diagnosis, significantly reducing the treatment window and survival benefits. Previous literature has indicated that approximately 70% of lung cancer patients are already in the advanced stage at the time of diagnosis, and about one-third of these patients die within 3 months of receiving the diagnosis (5). Hence, it is important to identify lung cancer patients at high risk of rapid progression to initiate timely treatment.

This study enrolled 1693 lung cancer patients from a retrospective cohort of 39799 individuals, to analyze the key risk factors of fast-growing lung cancers. It aims to provide evidence-based insights for early identification of high-risk patients, optimizing treatment timing, and intervention strategies in clinical practice.

## Methods

### Study design and population

**Figure 1:**
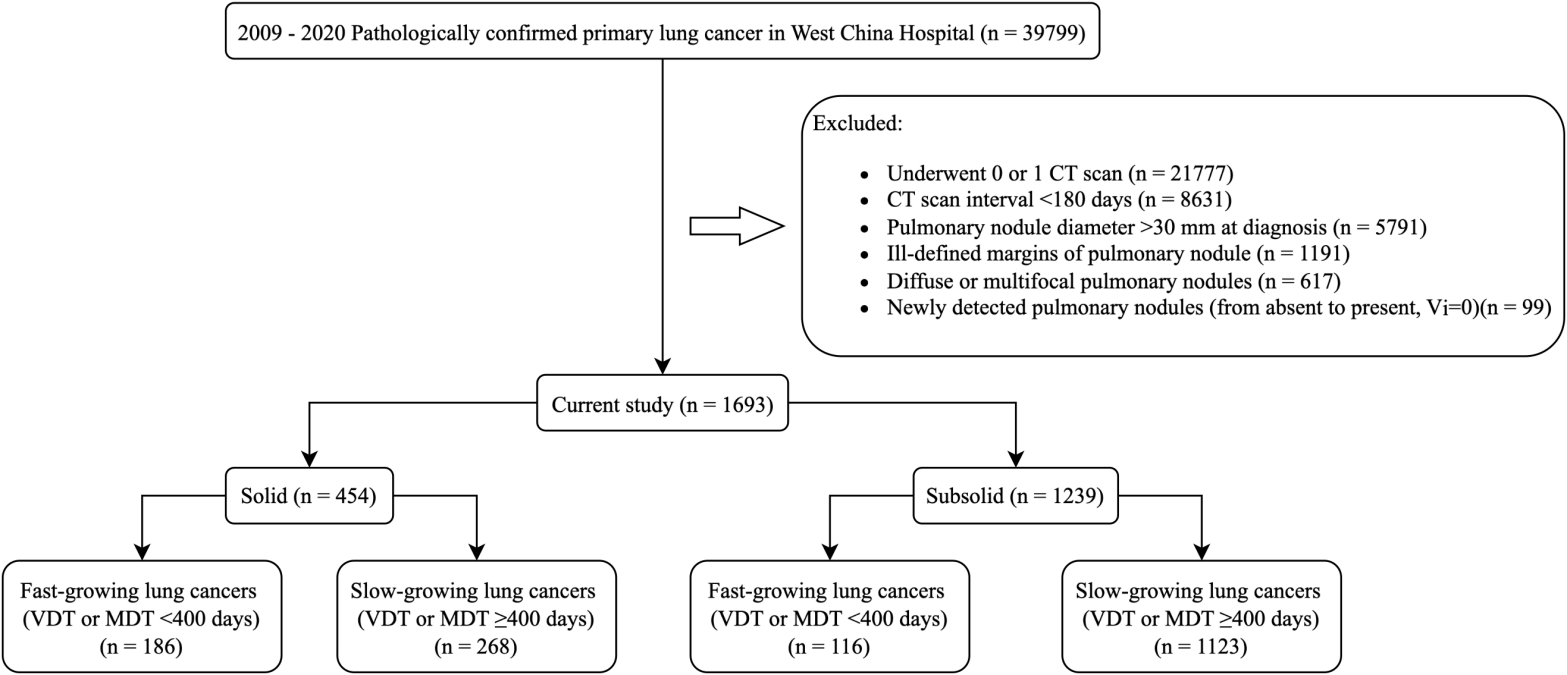
Study flowchart. CT, computed tomography; VDT, volume doubling time; MDT, mass doubling time.

This study screened research subjects from a clinical cohort of patients with pathologically diagnosed primary lung cancer at West China Hospital, Sichuan University, from 2009 to 2020. The exclusion criteria were as follows (1): Underwent 0 or 1 CT scan; (2) CT scan interval<180 days; (3) Pulmonary nodule diameter >30 mm at diagnosis; (4) Ill-defined margins of pulmonary nodule; (5) Diffuse or multifocal pulmonary nodules; (6) Newly detected pulmonary nodules (from absent to present, V1 = 0). Among the total of 39,799 patients, 1,693 patients met the criteria and were included in this study. The study flowchart is presented in **Figure 1**. This study was approved by the Biomedical Ethics Review Committee of West China Hospital, Sichuan University. The requirement for informed consent was waived due to the retrospective nature of the study.

### Clinical, pathological, genetic, and imaging data collection

For the included study subjects, the following clinical, pathological, and imaging data were retrieved from the Hospital Information System (HIS) of West China Hospital, Sichuan University. Demographic characteristics included age (years), sex (male, female), smoking history (ever smoker, never smoker), personal history of malignancy (positive, negative), family history of malignancy (positive, negative), methods of nodule detection (annual screening detected, respiratory symptoms, comorbid conditions), diagnostic methods (surgical biopsy, CT-guided PTNB, EBUS-TBNA, pleural fluid cytology, metastatic lesion biopsy), treatments (surgical interventions, non-surgical therapies), and time of pathological diagnosis. Collect the results of the most recent laboratory tumor marker tests before the patient’s diagnosis, including carcinoembryonic antigen (CEA, ng/ml), cytokeratin 19 (CYFRA 21-1, ng/ml), and neuron-specific enolase (NSE, ng/ml). Collect the results of the most recent pulmonary function test before the patient’s diagnosis, focusing on the ratio of forced expiratory volume in one second to forced vital capacity (FEV_1_/FVC). Spirometry-defined COPD was defined as FEV_1_/FVC<70% after bronchodilator administration, in accordance with GOLD (Global Initiative for Chronic Obstructive Lung Disease) recommendations. The pathological data includes pathological type (precursor glandular lesions, adenocarcinoma, squamous cell carcinoma, small cell carcinoma, other malignant neoplasms), T category (1–4), N category (0, 1, 2, 3), and M category (0, 1). Among 128 patients, 56-gene panel data for lung cancer were collected, which were categorized as wild-type and mutant-type (0, 1).

CT images from the initial detection of the lesions and the last preoperative CT images (with an interval of at least 180 days between the two CT examinations) were obtained. This study utilized the three-dimensional reconstruction software IQQA-Chest (EDDA Technology, Princeton Junction, NJ) research platform to export the required DICOM images from the PACS system of West China Hospital, Sichuan University. Subsequently, the open-source Pyradiomics platform was used to extract radiomics data, including volume and density, for each lung cancer from both the initial and last CT images to calculate volume/mass doubling time (VDT/MDT). Besides, the radiological features of lung cancers were recorded, including diameter (mm), location (upper lobe, non-upper lobe) and density (solid, subsolid). We evaluated the overall size of subsolid lung cancers. Solid lung cancers were defined as increased density in the pulmonary parenchyma where the edges of bronchi and blood vessels within the lesion are no longer identifiable. Subsolid lung cancers contained ground-glass opacity, which refer to increased density in the pulmonary parenchyma where the edges of bronchi and blood vessels within the lesion are still identifiable (6, 7).

### Definition of fast-growing lung cancers

Previous literature indicates that tumor growth follows an exponential pattern, and based on the exponential growth model, the time required for tumor doubling can be calculated (8). This study evaluates the progression rate of lung cancer through VDT (Volume Doubling Time, the time required for the volume of the tumor to double) and MDT (Mass Doubling Time, the time required for the mass of the tumor to double). The formula to calculate VDT/MDT is:


$$VDT/MDT\;=\;\frac{T\;\times \log2}{\log\left(\frac{X_{f}}{X_{i}}\right)}$$

In this formula, T represents the time interval in days between the two chest CT scans, X_f_ is the volume/mass of the pulmonary nodule on the last CT scan before surgery in cubic millimeters (mm^3^) or in milligrams (mg), and X_i_ is the volume/mass of the pulmonary nodule on the first CT scan when it was first detected in cubic millimeters (mm^3^) or in milligrams (mg) (9). Due to the approximate linear relationship between CT value and physical density (10), the calculation formula for mass M is:


$$M\;=\;V\;\times \;\left[\left(A_{mean}+1000\right)\times 0.001\right]$$

**Figure 2:**
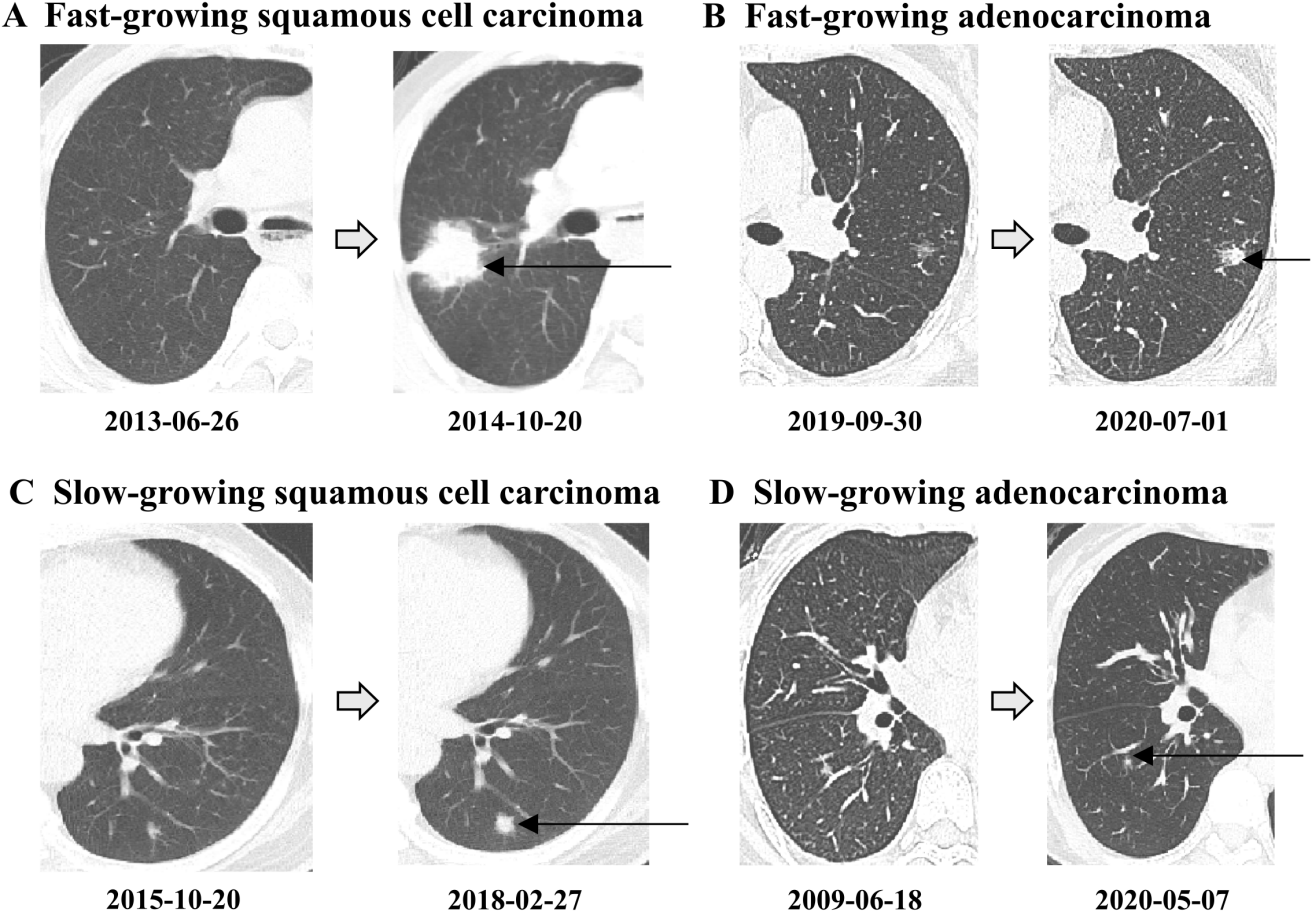
Examples of fast-growing and slow-growing lung cancers diagnosed by surgical histopathology. **(A)** Squamous cell carcinoma of the right upper lobe, postoperative pathological stage: Stage IIIA (T_2b_N_2a_M_0_), volume doubling time (VDT): 67 days. **(B)** Adenocarcinoma of the left upper lobe, postoperative pathological stage: Stage I A1 (T_1a_N_0_M_0_), VDT: 283 days. **(C)** Squamous cell carcinoma of the left lower lobe, postoperative pathological stage: Stage I A2 (T_1b_N_0_M_0_), VDT, 422 days. **(D)** Adenocarcinoma of the right lower lobe, postoperative pathological stage: Stage I A1 (T_1a_N_0_M_0_), VDT: 6346 days.

Here, V represents the volume of the pulmonary nodule, measured in cubic millimeters (mm^3^); and A_mean_ denotes the mean attenuation value, measured in Hounsfield units (Hu). Based on previous literature, when the VDT or MDT is less than 400 days, it is defined as fast-growing lung cancer; otherwise, it is defined as slow-growing lung cancer (11). In **Figure 2**, we have selected and displayed the CT images and volume doubling times of four typical cases, which are: A) Fast-growing squamous cell carcinoma, B) Fast-growing adenocarcinoma, C) Slow-growing squamous cell carcinoma, and D) Slow-growing adenocarcinoma.

### Data analysis

Categorical variables are presented as frequencies and percentages, and intergroup differences were assessed using Pearson’s chi-squared test with simulated p-values based on 10,000 Monte Carlo replicates. Continuous variables are expressed as median and interquartile range, and intergroup differences are evaluated by the Wilcoxon rank sum test. Multivariate logistic regression model was used to analyze the risk factors for fast-growing lung cancer. The data analysis of this study utilized the open-source R (12) v4.5.1 and the following key R packages: data preprocessing was performed with tidyverse (13) v2.0.0, the baseline characteristics table was generated using gtsummary (14) v2.4.0, multiple imputation for missing values was conducted with mice (15) v3.18.0, LASSO regression for variable selection was carried out using glmnet (16) v4.1-10, the forest plot for the multivariate logistic regression was created with forestploter (17) v1.1.3, and the gene heatmap was drawn with ComplexHeatmap (18) v2.24.1. A P-value less than 0.05 was considered statistically significant.

## Results

### Patients characteristics

From 2009 to 2020, among the 39,799 cases of lung cancer pathologically confirmed in West China Hospital, we identified 1,693 patients who met the inclusion and exclusion criteria. Among the 38,106 excluded patients, 21,777 underwent 0 or 1 CT scan; 8,631 had a time interval of less than 180 days between the initial CT scan identifying the pulmonary nodule and the last preoperative CT scan; 5,791 had a pulmonary nodule diameter greater than 30 mm at pathological diagnosis; 1,191 had ill-defined nodule margins on CT; 617 had diffuse or multifocal pulmonary nodules; and 99 had newly detected nodules (volume doubling time could not be calculated) (**Figure 1**).

In this study, which included 1,693 patients (median [IQR] age at pathological diagnosis 56 [48-65] years; 37% male; 21% with a history of smoking; 18% with rapid growth), 454 cases (27%) presented with solid density (median [IQR] age at pathological diagnosis 62 [54-69] years; 53% male; 37% with a history of smoking; 41% with rapid growth), and 1,239 cases (73%) presented with subsolid density (median [IQR] age at pathological diagnosis 54 [46-63] years; 31% male; 15% with a history of smoking; 9.4% with rapid growth). Among the overall population, 196 patients (12%) had a personal history of cancer, with 55 cases in the solid density group and 141 cases in the subsolid density group; 292 patients (17%) had a family history of cancer, with 68 cases in the solid density group and 224 cases in the subsolid density group (**Table 1**). Additionally, the majority of patients were diagnosed with pulmonary nodules through annual screening (69%), diagnosed via surgical biopsy (93%), and treated with surgery (94%). 146 patients (12%) had chronic obstructive pulmonary disease as defined by pulmonary function tests (Spirometry-defined COPD). Most pulmonary nodules were located in the upper lobe (62%), the predominant pathological type was adenocarcinoma (91%), most had a T stage of T_1_ (86%), an N stage of N_0_ (92%), an M stage of M_0_ (95%). The baseline characteristics of the overall population and stratified populations are presented in **Table 1**.

**Table 1:** Baseline characteristics of the population overall and stratified by density.

Variables	Overall n = 1693(100%)^1^	Density	
		Solid n = 454 (27%)	Subsolid n = 1,239 (73%)
Growth rate			
Fast (VDT/MDT<400d)	302 (18%)	186 (41%)	116 (9.4%)
Slow (VDT/MDT≥400d)	1,391 (82%)	268 (59%)	1,123 (91%)
Age, years	56(48-65)	62(54-69)	54(46-63)
Sex			
Male	627 (37%)	239 (53%)	388 (31%)
Female	1,066 (63%)	215 (47%)	851 (69%)
Smoking history			
Ever smoker	352 (21%)	168 (37%)	184 (15%)
Never smoker	1,341 (79%)	286 (63%)	1,055 (85%)
Personal history of malignancy			
Positive	196 (12%)	55 (12%)	141 (11%)
Negative	1,497 (88%)	399 (88%)	1,098 (89%)
Family history of malignancy			
Positive	292 (17%)	68 (15%)	224 (18%)
Negative	1,401 (83%)	386 (85%)	1,015 (82%)
Methods of nodule detection			
Annual screening detected	1,174 (69%)	260 (57%)	914 (74%)
Respiratory symptoms	346 (20%)	144 (32%)	202 (16%)
Comorbid conditions	173 (10%)	50 (11%)	123 (9.9%)
Diagnostic methods			
Surgical biopsy	1,577 (93%)	350 (77%)	1,227 (99%)
CT-guided PTNB^2^	78 (4.6%)	70 (15%)	8 (0.6%)
EBUS-TBNA^3^	26 (1.5%)	24 (5.3%)	2 (0.2%)
Pleural fluid cytology	5 (0.3%)	5 (1.1%)	0 (0%)
Metastatic lesion biopsy	7 (0.4%)	5 (1.1%)	2 (0.2%)
Treatments			
Surgical interventions	1,583 (94%)	354 (78%)	1,229 (99%)
Non-surgical therapies	110 (6.5%)	100 (22%)	10 (0.8%)
CEA, ng/mL	1.76(1.19-2.71)	2(2-4)	1.58(1.11-2.40)
CYFRA 21-1, ng/mL	2.10(1.56-2.80)	2.32(1.82-3.36)	2.01(1.51-2.62)
NSE, ng/mL	11.2(9.6-13.3)	11.8(9.9-14.7)	11.1(9.6-13.0)
Spirometry-defined COPD			
Positive	146 (12%)	71 (22%)	75 (8.2%)
Negative	1091 (88%)	253 (78%)	838 (92%)
Maximum diameter, mm	10.0(7.0-15.0)	13(10-19)	9.0(7.0-13.0)
Lung lobe			
Upper lobe	1,044 (62%)	213 (47%)	831 (67%)
Non-upper lobe	649 (38%)	241 (53%)	408 (33%)
Pathological type			
Precursor glandular lesions	70 (4.1%)	8 (1.8%)	62 (5.0%)
Adenocarcinoma	1,543 (91%)	374 (82%)	1,169 (94%)
Squamous cell carcinoma	48 (2.8%)	48 (11%)	0 (0%)
Small cell carcinoma	7 (0.4%)	7 (1.5%)	0 (0%)
Other malignant neoplasms	25 (1.5%)	17 (3.7%)	8 (0.6%)
T category			
1	1,397 (86%)	280 (63%)	1,117 (95%)
2	186 (11%)	129 (29%)	57 (4.8%)
3	17 (1.0%)	17 (3.8%)	0 (0%)
4	23 (1.4%)	20 (4.5%)	3 (0.3%)
N category			
0	1,489 (92%)	331 (74%)	1,158 (98%)
1	24 (1.5%)	23 (5.2%)	1 (<0.1%)
2	91 (5.6%)	77 (17%)	14 (1.2%)
3	19 (1.2%)	15 (3.4%)	4 (0.3%)
M category			
0	1,549 (95%)	384 (86%)	1,165 (99%)
1	74 (4.6%)	62 (14%)	12 (1.0%)

^1^n (%); Median(Q1-Q3). ^2^PTNB: percutaneous transthoracic needle biopsy. ^3^EBUS-TBNA: endobronchial ultrasound-guided transbronchial needle aspiration. Missing values: CEA, 12.7%; CYFRA 21-1, 16.7%; NSE, 19.7%; Spirometry-defined COPD, 26.9%; T, N, M, 4.1%.

In the univariate analysis of the overall population, there were statistically significant differences between the fast-growing group and the slow-growing group in terms of age, gender, smoking history, density and maximum diameter of pulmonary nodules, carcinoembryonic antigen (CEA) levels, and cytokeratin 19 (CYFRA 21-1) levels (P-value less than 0.001). However, after stratifying by the density of pulmonary nodules, only gender, smoking history and CEA maintained statistically significant differences between the fast-growing and slow-growing groups. Within the solid nodule subgroup, the P-values for age, gender, smoking history, CEA, CYFRA 21-1, and the lobe of the lung where the nodule was located were less than 0.05. Within the subsolid nodule subgroup, the P-values for gender, smoking history, CEA, and the maximum diameter of the nodule were less than 0.05. The characteristics of participants for the growth rate of lung cancer stratified by density are presented in **Table 2**. Although personal history of malignancy and family history of malignancy did not show statistically significant differences between the fast-growing and slow-growing groups in the univariate analysis, based on medical professional knowledge, we still included them in the multivariate analysis.

**Table 2:** Characteristics of participants for the growth rate of lung cancer stratified by density.

	Overall (n = 1693)			Solid (n = 454)			Subsolid (n = 1239)		
Variables	Fast (n = 302)	Slow (n = 1391)	P-value^2^	Fast (n = 186)	Slow (n = 268)	P-value	Fast (n = 116)	Slow (n = 1123)	P-value
Age, years	60(53-67)^1^	55(47-64)	<0.001	63(55-70)	61(53-67)	0.031	56(49-63)	54(46-63)	0.058
Sex			<0.001			<0.001			0.03
Male	185 (61%)	442 (32%)		138 (74%)	101 (38%)		47 (41%)	341 (30%)	
Female	117 (39%)	949 (68%)		48 (26%)	167 (62%)		69 (59%)	782 (70%)	
Smoking history			<0.001			<0.001			0.001
Ever smoker	140 (46%)	212 (15%)		110 (59%)	58 (22%)		30 (26%)	154 (14%)	
Never smoker	162 (54%)	1,179 (85%)		76 (41%)	210 (78%)		86 (74%)	969 (86%)	
Personal history of malignancy			0.075			0.14			0.4
Positive	44 (15%)	152 (11%)		28 (15%)	27 (10%)		16 (14%)	125 (11%)	
Negative	258 (85%)	1,239 (89%)		158 (85%)	241 (90%)		100 (86%)	998 (89%)	
Family history of malignancy			0.2			0.11			0.13
Positive	61 (20%)	231 (17%)		34 (18%)	34 (13%)		27 (23%)	197 (18%)	
Negative	241 (80%)	1,160 (83%)		152 (82%)	234 (87%)		89 (77%)	926 (82%)	
Methods of nodule detection			<0.001			0.008			0.7
Annual screening detected	178(59%)	996(72%)		90(48%)	170(63%)		88(76%)	826(74%)	
Respiratory symptoms	90(30%)	256(18%)		71(38%)	73(27%)		19(16%)	183(16%)	
Comorbid conditions	34(11%)	139(10%)		25(13%)	25(9.3%)		9(7.8%)	114(10%)	
CEA, ng/mL	2.25(1.42-3.79)	1.69(1.15-2.54)	<0.001	3(2-5)	2(1-4)	0.01	1.76(1.22-2.84)	1.57(1.10-2.33)	0.042
CYFRA 21-1, ng/mL	2.33(1.76-3.33)	2.07(1.54-2.67)	<0.001	2.64(1.96-3.97)	2.25(1.72-3.24)	0.003	2.02(1.47-2.98)	2.01(1.51-2.60)	0.5
NSE, ng/mL	11.4(9.8-13.3)	11.2(9.6-13.3)	0.2	11.7(10.1-15.0)	11.9(9.7-14.6)	0.6	11.0(9.5-12.6)	11.1(9.6-13.0)	0.4
Maximum diameter, mm	12.0(9.0-18.0)	10.0(7.0-14.0)	<0.001	14(10-20)	12(9-19)	0.2	10.5(7.5-15.0)	9.0(7.0-13.0)	0.012
Lung lobe			0.2			0.044			>0.9
Upper lobe	176 (58%)	868 (62%)		98 (53%)	115 (43%)		78 (67%)	753 (67%)	
Non-upper lobe	126 (42%)	523 (38%)		88 (47%)	153 (57%)		38 (33%)	370 (33%)	
T category			<0.001			<0.001			0.002
1	207(69%)	1190(90%)		103(55%)	177(68%)		104(90%)	1013(95%)	
2	68(23%)	118(8.9%)		58(31%)	71(27%)		10(8.6%)	47(4.4%)	
3	16(5.3%)	1(<0.1%)		16(8.6%)	1(0.4%)		0(0%)	0(0%)	
4	11(3.6%)	12(0.9%)		9(4.8%)	11(4.2%)		2(1.7%)	1(<0.1%)	
N category			<0.001			<0.001			0.001
0	228(75%)	1261(95%)		119(64%)	212(82%)		109(94%)	1049(99%)	
1	16(5.3%)	8(0.6%)		15(8.1%)	8(3.1%)		1(0.9%)	0(0%)	
2	49(16%)	42(3.2%)		45(24%)	32(12%)		4(3.4%)	10(0.9%)	
3	9(3.0%)	10(0.8%)		7(3.8%)	8(3.1%)		2(1.7%)	2(0.2%)	
M category			<0.001			0.6			0.004
0	269(89%)	1280(97%)		158(85%)	226(87%)		111(96%)	1054(99%)	
1	33(11%)	41(3.1%)		28 (15%)	34 (13%)		5(4.3%)	7(0.7%)	
Density			<0.001						
Solid	186 (62%)	268 (19%)							
Subsolid	116 (38%)	1,123 (81%)							

^1^Median(Q1-Q3); n (%). ^2^Wilcoxon rank sum test; Pearson’s Chi-squared test with simulated p-value (based on 10000 replicates). Missing values: CEA, 12.7%; CYFRA 21-1, 16.7%; NSE, 19.7%; T, N, M, 4.1%.

### Risk factors associated with fast-growing lung cancers

**Figure 3:**
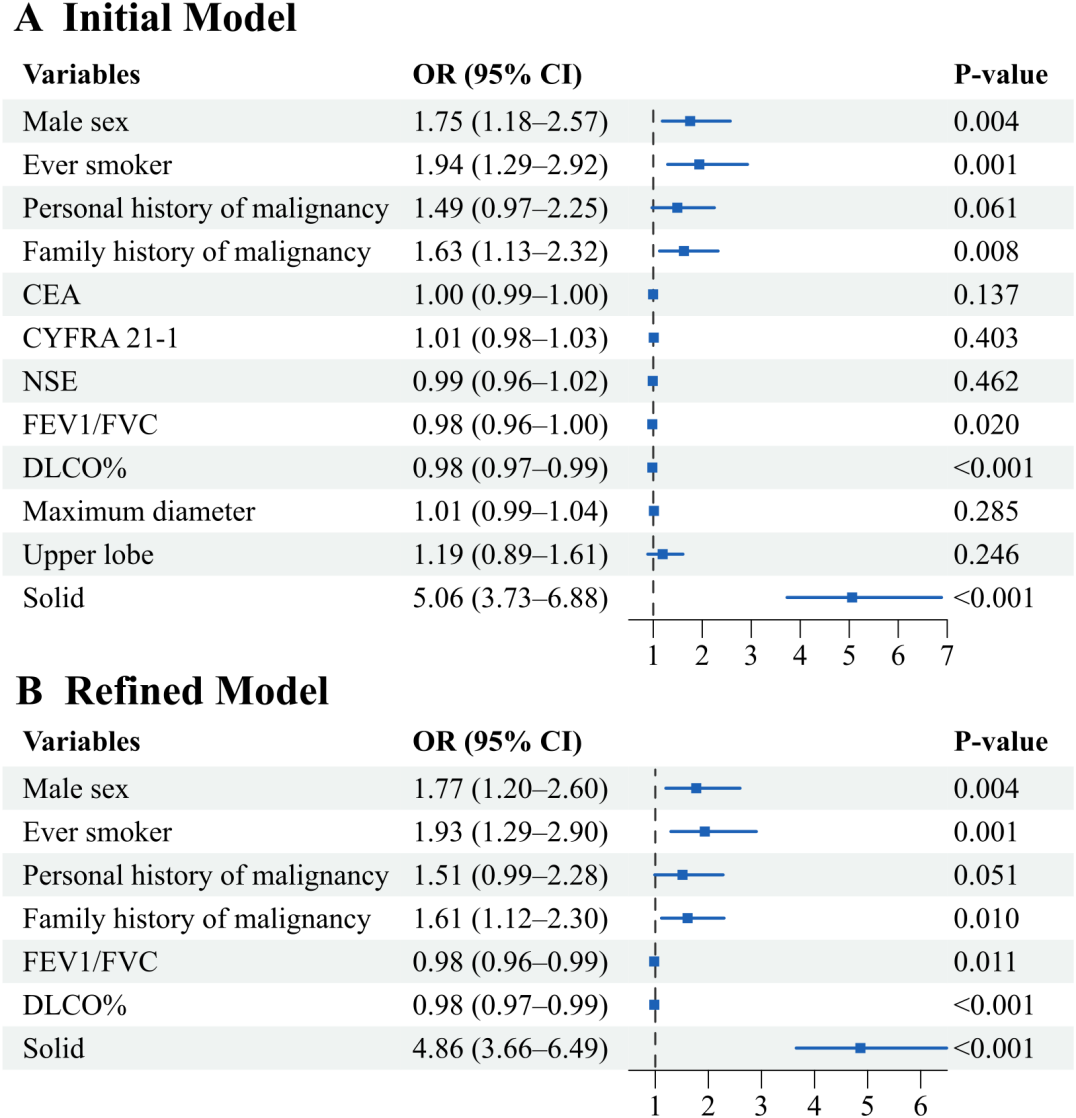
Odds ratios for risk factors independently associated with fast-growing lung cancers. **(A)** Forest plot of the initial model incorporating variables selected by LASSO regression supplemented with clinically relevant factors. **(B)** Forest plot of the refined parsimonious model. Odds ratios with 95% confidence intervals are shown. The vertical dashed line indicates the null effect (OR = 1).

In the multivariate logistic regression analysis, we first used LASSO to screen variables to establish an initial model. Subsequently, we removed most irrelevant variables to form a refined model. In the multivariate logistic regression analysis of the overall population, the density of lung cancers being solid was the largest risk factor, with an odds ratio (OR) of 4.86 and a 95% confidence interval (95% CI) of 3.66 – 6.49. Additionally, male sex (OR 1.77, 95% CI 1.20 – 2.60), smoking history (OR 1.93, 95% CI 1.29 – 2.90), and family history of malignancy (OR 1.61, 95% CI 1.12 – 2.30) were also risk factors for the rapid growth of lung cancer. Although the p-value for personal history of malignancy (OR 1.51, 95% CI 0.99 – 2.28) was marginally significant at 0.051, we still consider it to be associated with rapid growth and warranting attention. The odds ratios for risk factors independently associated with fast-growing lung cancers are presented in **Figure 3**.

**Figure 4:**
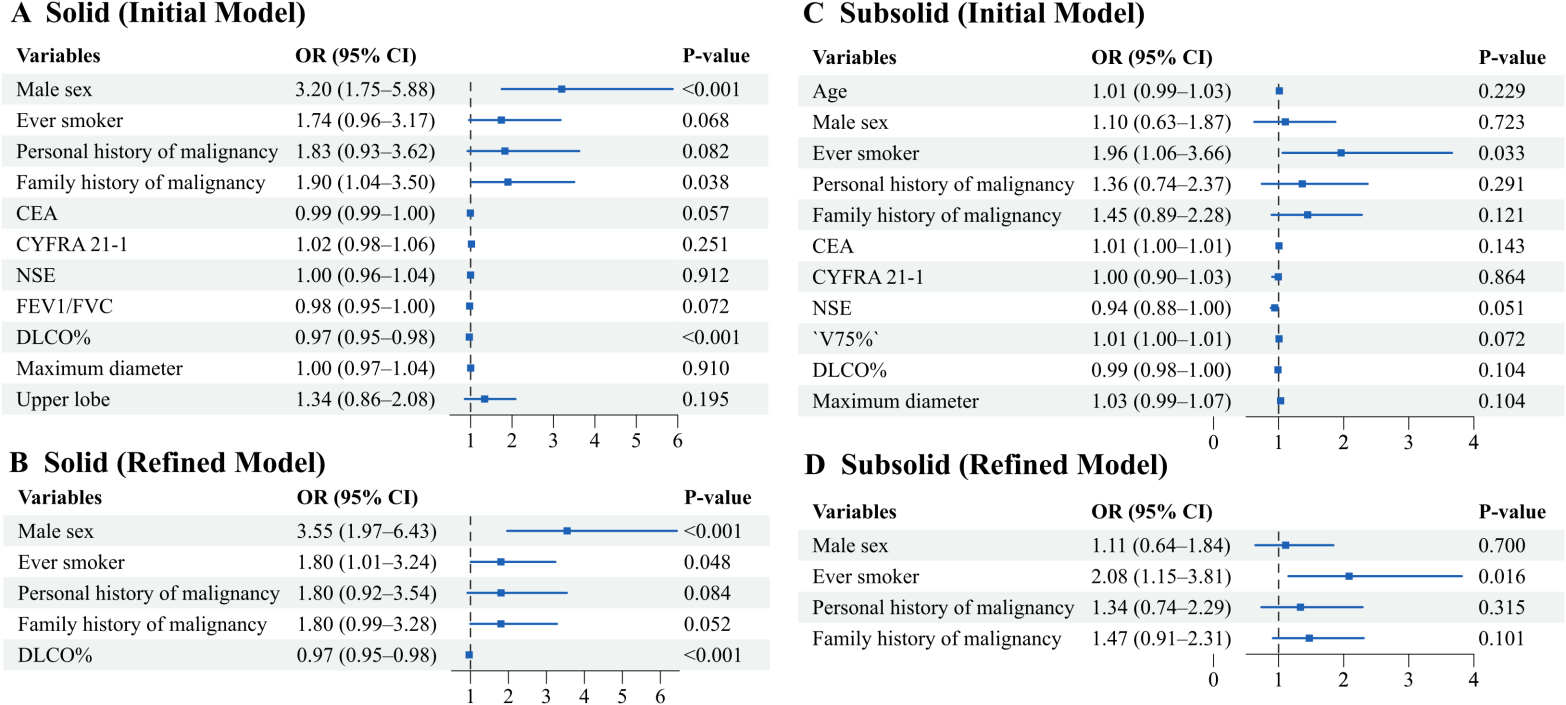
Odds ratios of risk factors independently associated with fast-growing lung cancers by density. **(A, B)** Solid. **(C, D)** Subsolid. Initial model: Forest plot of the initial model incorporating variables selected by LASSO regression supplemented with clinically relevant factors. Refined model: Forest plot of the refined parsimonious model. Odds ratios with 95% confidence intervals are shown. The vertical dashed line indicates the null effect (OR = 1).

In the subgroup of solid lung cancer, male sex (OR 3.55, 95% CI 1.97 - 6.43) and having a history of smoking (OR 1.80, 95% CI 1.01 - 3.24) were independent risk factors for the rapid growth of lung cancer. However, in the subsolid group, only a history of smoking (OR 2.08, 95% CI 1.15 - 3.81) remained a risk factor, while the influence of gender on the rapid growth of lung cancer was no longer significant. The odds ratios for risk factors independently associated with fast-growing lung cancers by density are presented in **Figure 4**.

**Figure 5:**
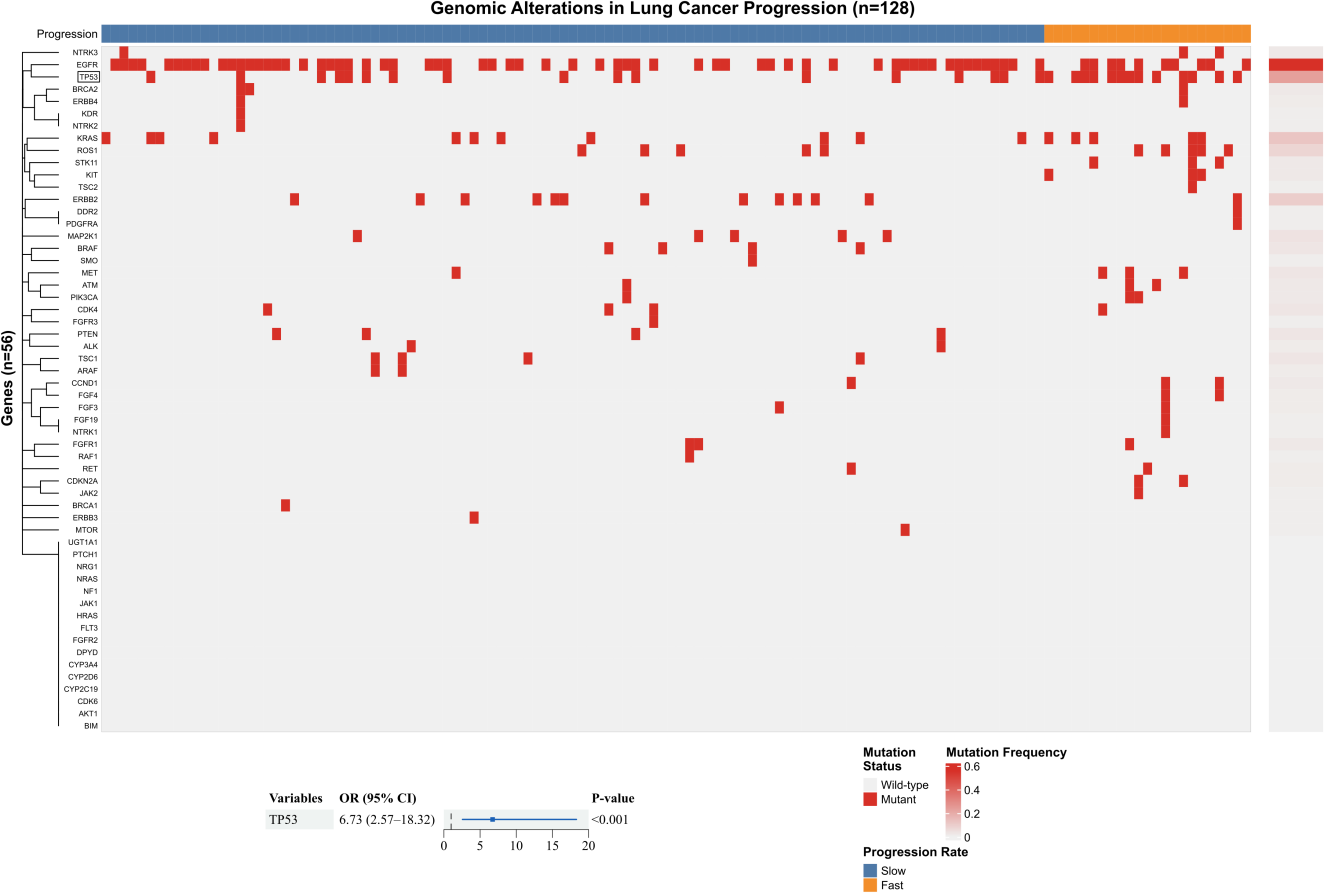
Genomic alterations and their association with rapid progression in lung cancer. Heatmap displaying mutation profiles of 56 cancer-related genes across 128 lung cancer patients, stratified by progression rate (Slow vs. Fast). Rows represent individual genes, columns represent patients. Mutation status is indicated in red (mutant) and gray (wild-type). Genes are ordered by mutation frequency (from highest to lowest) and clustered based on similarity of mutation patterns. The adjacent bar plot shows mutation frequency for each gene. Forest plot illustrating multivariate logistic regression analysis of genes significantly associated with rapid progression, identified through LASSO regularization. Odds ratios (OR) with 95% confidence intervals are shown for each significant genetic alteration. Reference line indicates no effect (OR = 1).

Among the 1693 patients, 128 had undergone lung cancer genetic testing using 56-gene panel. We conducted an analysis of risk factors for rapid lung cancer growth within this small subset. **Figure 5** presents a heatmap of the 56-gene panel for the 128 patients (23 with rapid growth, accounting for 18%; 105 with slow growth, accounting for 82%), and the only significant gene identified after LASSO screening (alpha=1, 1se set) was TP53. The odds ratio for TP53 mutation type was as high as 6.73, with a 95% confidence interval of 2.57 - 18.32. TP53 mutation type appears to be a potential risk factor for rapid lung cancer growth, but this finding is based on a small subset of patients. Validation in larger, independent cohorts is necessary before drawing definitive conclusions.

To assess potential confounding by methods of nodule detection (annual screening, respiratory symptoms, or comorbid conditions), we performed stratified analyses, with results presented in **Supplementary Figure 1**. Solid nodule was consistently associated with fast-growing across all detection pathways (OR: 4.45–10.49; all P<0.001), indicating that the detection method did not confound our main findings.

## Discussion

This study retrospectively analyzed 1693 patients who met the inclusion and exclusion criteria at West China Hospital from 2009 to 2020, revealing that the density of pulmonary nodules, gender, smoking history, personal and family history of malignancy and TP53 mutation type are independent risk factors for the rapid growth of lung cancer. It also pointed out that age, diameter, and location, as well as common clinical tumor markers, are difficult to differentiate fast-growing lung cancers from slow-growing ones.

In the analysis of the overall population, the presence of solid pulmonary nodule density was the greatest risk factor, with an odds ratio (95% confidence interval) of 4.86 (3.66 - 6.49), and the P-value was less than 0.001. Among the 302 cases of rapidly growing lung cancer, 62% were solid; whereas among the 1391 cases of slowly growing lung cancer, only 19% were solid. To control for potential confounding effects and investigate whether the influence of other risk factors on growth rate differs under different lung nodule densities, we divided the overall population into two subgroups: solid and subsolid.

Tumor stage correlated with growth rate, but patterns diverged by density. In solid lung cancers, fast growth linked to higher T_2_–T_4_ (44.4% vs. 31.6%) and N_1_–N_3_ (35.9% vs. 18.2%) categories (P<0.001), not M_1_ (P = 0.6). In subsolid lung cancers, fast growth also associated with advanced T_2_–T_4_ (10.3% vs. 4.5%; P = 0.002) and N_1_–N_3_ (6.0% vs. 1.1%; P = 0.001). Thus, rapid growth signals aggressiveness, but its implication differs: in solid ones it reflects established advanced disease, whereas in subsolid ones it indicates accelerated early-stage progression. Density stratification is therefore essential when interpreting growth dynamics.

In both the overall population and the two subgroups, a history of smoking was a significant independent risk factor for rapidly growing lung cancer. Compared to non-smokers, the OR (95% CI) values for a positive smoking history were 1.93 (1.29 - 2.90) in the overall population, 1.80 (1.01 - 3.24) in the solid subgroup, and 2.08 (1.15 - 3.81) in the subsolid subgroup. The odds ratio remained largely unchanged before and after stratification, indicating that regardless of whether the imaging findings suggest solid or subsolid nodules, refraining from smoking can reduce the risk of potential rapid growth in lung cancer screening. According to previous literature, in 2023, 32.18% of individuals in the China Kadoorie Biobank (CKB) cohort were ever smokers (19), indicating that there is still great potential to reduce mortality and economic losses caused by fast-growing lung cancer by advocating for never smoking.

Patient sex difference showed a statistically significant impact on the growth rate of lung cancer in the overall population, with an OR (95%CI) value of 1.77 (1.20 - 2.60) and a P-value of 0.004. After stratification, males in the solid subgroup had an OR (95%CI) value of 3.55 (1.97 - 6.43) compared to females, with a P-value less than 0.001, indicating that male sex is a prominent risk factor when the nodules are solid. However, in the subsolid subgroup, the OR (95%CI) value was 1.11 (0.64 - 1.84) with a P-value of 0.700, suggesting that sex is a non-significant factor when the nodules are subsolid. This difference before and after stratification indicates that for patients with solid pulmonary nodules, special attention should be paid to males, as they have a higher risk of rapid growth in potential lung cancer, necessitating more active follow-up and intervention. Previous literature has mentioned that there are genetic differences between males and females in the occurrence and development of lung cancer (20). The distinction in genetic susceptibility between sexes may be one of the reasons why solid lung cancer grows faster in males, but further research is needed to confirm the specific mechanisms.

A positive personal or family history of malignancy also emerged as an independent risk factor for rapid lung cancer growth in the overall population, with OR (95%CI) values of 1.51 (0.99 - 2.28) and 1.61 (1.12 - 2.30), and P-values of 0.051 and 0.010, respectively. However, after stratification, this statistical significance disappeared: within the solid subgroup, the OR (95%CI) values were 1.80 (0.92 - 3.54) and 1.80 (0.99 - 3.28), with P-values of 0.084 and 0.052, respectively; within the subsolid subgroup, the OR (95%CI) values were 1.34 (0.74 - 2.29) and 1.47 (0.91 - 2.31), with P-values of 0.315 and 0.101, respectively. The emergence of this phenomenon is suspected to be due to the fact that stratification has reduced the sample size of each subgroup, resulting in decreased statistical power, rendering it no longer significant. Considering that a personal or family history of malignancy may indicate a higher genetic susceptibility or exposure to a lifestyle conducive to carcinogenesis, it is reasonable to believe that their effects on rapid lung cancer growth are both positive.

The association between TP53 mutation and rapid tumor growth, while intriguing, should be interpreted with caution due to the limited sample size and wide confidence intervals. This finding represents a hypothesis-generating observation that warrants validation in larger studies. LASSO suggested that its role surpasses the other 55 genes, potentially being one of the sources of the biological mechanism underlying rapid growth. Previous studies have indicated that during the evolution of lung adenocarcinoma, p53 induces the differentiation of alveolar type 1 epithelial cells through direct DNA binding, chromatin remodeling, and the induction of specific genes. The inactivation of p53 leads to the improper persistence of transit-amplifying cancer cells in a transient intermediate state, similar to the AT2 to AT1 cell differentiation during alveolar injury repair (21). p53 also plays a role in guiding alveolar regeneration after injury by regulating AT2 cell self-renewal and promoting the differentiation of transit cells into AT1 cells. A real-world retrospective cohort study also reported a significant association between TP53 mutations and male gender, adenocarcinoma differentiation, smoking history, PD-L1 tumor proportion score, and tumor mutation burden level (22). The p53 protein is encoded by the TP53 gene, therefore mutations in TP53 may result in accelerated tumor growth, warranting clinical attention. Previous studies have suggested that EGFR/TP53 co-mutations may be associated with poorer prognosis (23, 24). We identified 20 such cases, 5 of which were fast-growing lung cancers. However, in our preliminary logistic regression analysis, this co-mutation did not show a significant association with rapid growth. This may be due to the limited sample size, warranting validation in larger cohorts. KRAS mutations are less common in Chinese lung cancer patients (approximately 12.1%) compared to Western populations (approximately 32.9%) (25, 26), which may explain the rarity of KRAS mutations in our cohort. This epidemiological difference should be considered when interpreting our findings in the context of previous studies that reported KRAS mutations as associated with faster growth in Western populations.

It is noteworthy that regardless of whether in the overall population or the stratified subgroups, patient age, diameter and location, pulmonary function indicators, and the levels of three clinically common tumor markers (CEA, CYFRA 21-1, NSE) were not found to have an impact on the rapid growth of lung cancer in the multivariate logistic regression analysis. Even if the P-value of some individual factors was less than 0.05, the effect size was very small, making it difficult to provide valuable guidance for clinical decision-making. A 2022 review article pointed out that the role of traditional biomarkers is largely limited by the spatial and temporal heterogeneity of tumors, whereas new markers such as CTCs (Circulating Tumor Cells), ctDNA, and exosomes detected by liquid biopsies seem promising in overcoming this heterogeneity. Future research is needed to investigate whether these novel tumor markers can assist in distinguishing rapidly growing lung cancer (27).

This study has some limitations. Firstly, since not all patients underwent pulmonary function testing, approximately 26.9% of the pulmonary function test data were missing. After multiple imputation to fill in the missing values, multivariate regression analysis may have introduced bias. In our study, the impact of pulmonary function indicators such as FEV_1_/FVC and DLCO% on the growth rate of lung cancer was extremely weak. However, previous literature has indicated that lung cancer and chronic obstructive pulmonary disease (COPD) often occur simultaneously, and individuals with COPD have a higher risk of developing lung cancer. The potential mechanisms for this increased risk may include genomic, immune, and microenvironmental dysregulation (28). Additionally, a small sample study suggested that the median volume doubling time for lung cancer with COPD was 126.1 days (29), which may also imply that COPD is one of the risk factors for rapid lung cancer growth. Larger sample sizes are needed in the future to confirm whether COPD has a significant impact on the rapid growth of lung cancer. Secondly, the smoking history data we collected was binary, only categorizing individuals as either ever smokers or never smokers. However, previous literature has shown that the incidence, progression, and prognosis of lung cancer are associated with the amount of smoking in pack-years (30). Additionally, research has indicated that for COPD patients who smoke fewer than 30 pack-years, reducing smoking and quitting can decrease the risk of developing lung cancer (31). This further highlights the potential importance of the specific amount of smoking. In the future, more detailed smoking history information is needed to address this limitation. Thirdly, the timing of the ‘last pre-diagnostic CT’ was often dictated by clinical decision-making which may have introduced management-related bias. Shorter observation windows may amplify the impact of volumetric measurement errors on VDT calculations. As noted in pulmonary nodule guidelines, monitoring the increase in the solid component of subsolid nodules is also important. We plan to collect and analyze this specific information in our future studies. Finally, the majority of patients admitted to West China Hospital are Chinese, and the population included in this study may have homogeneity in terms of ethnicity. Therefore, the conclusions may not be applicable to other ethnic groups.

## Conclusions

This study explored the risk factors associated with the fast-growing lung cancers, emphasizing the need for attention and early intervention in individuals with solid nodules, males, smokers, and those with TP53 mutations. Additionally, it clarified that age, diameter, location, and traditional tumor markers have no predictive significance for fast-growing lung cancer.

## Data Availability

The raw data supporting the conclusions of this article will be made available by the authors, without undue reservation.

## Supplementary material

Supplementary Figure 1 Odds ratios of risk factors independently associated with fast-growing lung cancers by methods of detection. **(A)** Annual screening detected. **(B)** Respiratory symptoms. **(C)** Comorbid conditions. Odds ratios with 95% confidence intervals are shown. The vertical dashed line indicates the null effect (OR = 1).

## References

[1] Bray F Laversanne M Sung H Ferlay J Siegel RL Soerjomataram I . Global cancer statistics 2022: GLOBOCAN estimates of incidence and mortality worldwide for 36 cancers in 185 countries. CA A Cancer J Clin. (2024) 74:229–63. doi: 10.3322/caac.21834, PMID: 38572751

[2] Han B Zheng R Zeng H Wang S Sun K Chen R . Cancer incidence and mortality in China, 2022. J Natl Cancer Center. (2024) 4:47–53. doi: 10.1016/j.jncc.2024.01.006, PMID: 39036382 PMC11256708

[3] Corby G Barclay NL Tan EH Burn E Delmestri A Duarte-Salles T . Incidence, prevalence, and survival of lung cancer in the United Kingdom from 2000–2021: a population-based cohort study. Transl Lung Cancer Res. (2024) 13:2187–201. doi: 10.21037/tlcr-24-241, PMID: 39430337 PMC11484731

[4] Loomis D Guha N Hall AL Straif K . Identifying occupational carcinogens: an update from the IARC Monographs. Occup Environ Med. (2018) 75:593–603. doi: 10.1136/oemed-2017-104944, PMID: 29769352 PMC6204931

[5] Huang S Tang O Zheng X Li H Wu Y Yang L . Effectiveness of smoking cessation on the high-risk population of lung cancer with early screening: a systematic review and meta-analysis of randomized controlled trials until January 2022. Arch Public Health. (2023) 81:101. doi: 10.1186/s13690-023-01111-5, PMID: 37268972 PMC10239152

[6] Mazzone PJ Lam L . Evaluating the patient with a pulmonary nodule: A review. JAMA. (2022) 327:264. doi: 10.1001/jama.2021.24287, PMID: 35040882

[7] National Institutes for Food and Drug Control Cardio-thoracic Working Group Chinese Society of Radiology Chinese Medical Association . Expert consensus on the rule and quality control of pulmonary nodule annotation based on thoracic CT. Chin J Radiol. (2019) 53:9–15. doi: 10.3760/cma.j.issn.1005-1201.2019.01.004, PMID: 40668938

[8] Heuvelmans MA Vliegenthart R De Koning HJ Groen HJM Van Putten MJAM Yousaf-Khan U . Quantification of growth patterns of screen-detected lung cancers: The NELSON study. Lung Cancer. (2017) 108:48–54. doi: 10.1016/j.lungcan.2017.02.021, PMID: 28625647

[9] Schwartz M . A biomathematical approach to clinical tumor growth. Cancer. (1961) 14:1272–94. doi: 10.1002/1097-0142(196111/12)14:6<1272::AID-CNCR2820140618>3.0.CO;2-H 13909709

[10] Mull R . Mass estimates by computed tomography: physical density from CT numbers. Am J Roentgenol. (1984) 143:1101–4. doi: 10.2214/ajr.143.5.1101, PMID: 6333158

[11] Lu H Mu W Balagurunathan Y Qi J Abdalah MA Garcia AL . Multi-window CT based Radiomic signatures in differentiating indolent versus aggressive lung cancers in the National Lung Screening Trial: a retrospective study. Cancer Imaging. (2019) 19:45. doi: 10.1186/s40644-019-0232-6, PMID: 31253194 PMC6599273

[12] R Core Team . R: A language and environment for statistical computing. (2025). Available online at: https://www.r-project.org/ (Accessed September 1, 2025).

[13] Wickham H Averick M Bryan J Chang W McGowan L François R . Welcome to the tidyverse. JOSS. (2019) 4:1686. doi: 10.21105/joss.01686

[14] Sjoberg DD Whiting K Curry M Lavery JA Larmarange J . Reproducible summary tables with the gtsummary package. R J. (2021) 13:570. doi: 10.32614/RJ-2021-053, PMID: 41351832

[15] Buuren SV Groothuis-Oudshoorn K . mice : multivariate imputation by chained equations in *R*. J Stat Soft. (2011) 45:1–67. doi: 10.18637/jss.v045.i03

[16] Friedman J Hastie T Tibshirani R . Regularization paths for generalized linear models via coordinate descent. J Stat Soft. (2010) 33:1–22. doi: 10.18637/jss.v033.i01 PMC2929880 20808728

[17] Dayimu A . forestploter: create a flexible forest plot. (2025) R package version 1.1.3. Available online at: https://CRAN.R-project.org/package=forestploter (Accessed September 1, 2025).

[18] Gu Z . Complex heatmap visualization. iMeta. (2022) 1:e43. doi: 10.1002/imt2.43, PMID: 38868715 PMC10989952

[19] Ma Z Lv J Zhu M Yu C Ma H Jin G . Lung cancer risk score for ever and never smokers in China. Cancer Commun. (2023) 43:877–95. doi: 10.1002/cac2.12463, PMID: 37410540 PMC10397566

[20] Mederos N Friedlaender A Peters S Addeo A . Gender-specific aspects of epidemiology, molecular genetics and outcome: lung cancer. ESMO Open. (2020) 5:e000796. doi: 10.1136/esmoopen-2020-000796, PMID: 33148544 PMC7643520

[21] Kaiser AM Gatto A Hanson KJ Zhao RL Raj N Ozawa MG . p53 governs an AT1 differentiation programme in lung cancer suppression. Nature. (2023) 619:851–9. doi: 10.1038/s41586-023-06253-8, PMID: 37468633 PMC11288504

[22] Hao F Gu L Zhong D . TP53 mutation mapping in advanced non-small cell lung cancer: A real-world retrospective cohort study. Curr Oncol. (2022) 29:7411–9. doi: 10.3390/curroncol29100582, PMID: 36290859 PMC9599964

[23] Gallina FT Marinelli D Tajè R Visca P Cecere FL Landi L . TP53 co-mutations increase risk of recurrence in EGFR-mutated stage I lung adenocarcinoma. Eur J Cancer. (2025) 227:115622. doi: 10.1016/j.ejca.2025.115622, PMID: 40680467

[24] Skoulidis F Heymach JV . Co-occurring genomic alterations in non-small-cell lung cancer biology and therapy. Nat Rev Cancer. (2019) 19:495–509. doi: 10.1038/s41568-019-0179-8, PMID: 31406302 PMC7043073

[25] Chen H Huang D Lin G Yang X Zhuo M Chi Y . The prevalence and real‐world therapeutic analysis of Chinese patients with KRAS‐mutant non‐small cell lung cancer. Cancer Med. (2022) 11:3581–92. doi: 10.1002/cam4.4739, PMID: 35394121 PMC9554448

[26] Izumi M Suzumura T Ogawa K Matsumoto Y Sawa K Yoshimoto N . Differences in molecular epidemiology of lung cancer among ethnicities (asian vs. Caucasian). J Thorac Dis. (2020) 12:3776–84. doi: 10.21037/jtd.2019.08.61, PMID: 32802457 PMC7399397

[27] Li W Liu J-B Hou L-K Yu F Zhang J Wu W . Liquid biopsy in lung cancer: significance in diagnostics, prediction, and treatment monitoring. Mol Cancer. (2022) 21:25. doi: 10.1186/s12943-022-01505-z, PMID: 35057806 PMC8772097

[28] Forder A Zhuang R Souza VGP Brockley LJ Pewarchuk ME Telkar N . Mechanisms contributing to the comorbidity of COPD and lung cancer. IJMS. (2023) 24:2859. doi: 10.3390/ijms24032859, PMID: 36769181 PMC9918127

[29] Kim C Lee SM Choe J Chae EJ Do K-H Seo JB . Volume doubling time of lung cancer detected in idiopathic interstitial pneumonia: comparison with that in chronic obstructive pulmonary disease. Eur Radiol. (2018) 28:1402–9. doi: 10.1007/s00330-017-5091-6, PMID: 29038933

[30] Ban WH Yeo CD Han S Kang HS Park CK Kim JS . Impact of smoking amount on clinicopathological features and survival in non-small cell lung cancer. BMC Cancer. (2020) 20:848. doi: 10.1186/s12885-020-07358-3, PMID: 32883225 PMC7469911

[31] Shin SH Kim T Kim H Cho J Kang D Park HY . Impact of smoking reduction on lung cancer risk in patients with COPD who smoked fewer than 30 pack-years: a nationwide population-based cohort study. Respir Res. (2024) 25:133. doi: 10.1186/s12931-024-02741-1, PMID: 38500143 PMC10949658

