# Supplementary figures and images for: Risk factors for fast-growing lung cancers detected on chest CT: a retrospective cohort study
Source: Front Oncol. 2026 Mar 20;16:1789510. doi: 10.3389/fonc.2026.1789510 (PMC13046516; doi:10.3389/fonc.2026.1789510)

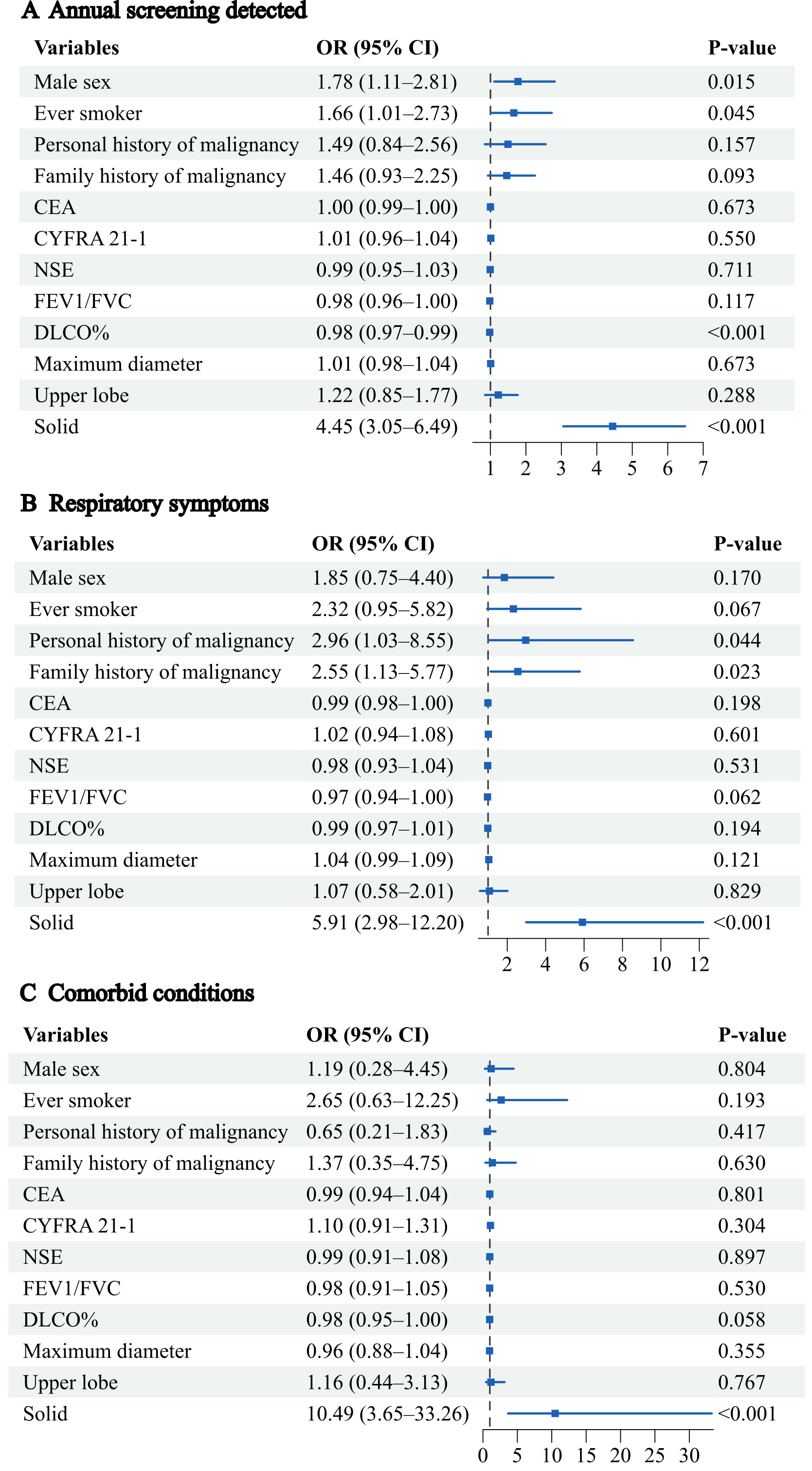

Supplement: Supplementary Figure 1 — Odds ratios of risk factors independently associated with fast-growing lung cancers by methods of detection. (A) Annual screening detected. (B) Respiratory symptoms. (C) Comorbid conditions. Odds ratios with 95% confidence intervals are shown. The vertical dashed line indicates the null effect (OR = 1). [file Image1.tiff]
